# Testing normative and self-appraisal feedback in an online slot-machine pop-up in a real-world setting

**DOI:** 10.3389/fpsyg.2015.00339

**Published:** 2015-03-23

**Authors:** Michael M. Auer, Mark D. Griffiths

**Affiliations:** ^1^neccton ltd., London, UK; ^2^Gaming Research Unit, Psychology Division, Nottingham Trent University, Nottingham, UK

**Keywords:** online gambling, responsible gambling, online slot machines, pop-up messaging, normative feedback, ecological validity, behavioral tracking, health messaging

## Abstract

Over the last few years, there have been an increasing number of gaming operators that have incorporated on-screen pop-up messages while gamblers play on slot machines and/or online as one of a range of tools to help encourage responsible gambling. Coupled with this, there has also been an increase in empirical research into whether such pop-up messages are effective, particularly in laboratory settings. However, very few studies have been conducted on the utility of pop-up messages in real-world gambling settings. The present study investigated the effects of normative and self-appraisal feedback in a slot machine pop-up message compared to a simple (non-enhanced) pop-up message. The study was conducted in a real-world gambling environment by comparing the behavioral tracking data of two representative random samples of 800,000 gambling sessions (i.e., 1.6 million sessions in total) across two conditions (i.e., simple pop-up message versus an enhanced pop-up message). The results indicated that the additional normative and self-appraisal content doubled the number of gamblers who stopped playing after they received the enhanced pop-up message (1.39%) compared to the simple pop-up message (0.67%). The data suggest that pop-up messages influence only a small number of gamblers to cease long playing sessions and that enhanced messages are slightly more effective in helping gamblers to stop playing in-session.

## Introduction

The increasingly advanced technological environments of online gambling companies now allow for sophisticated ways of promoting responsible play among gamblers (Griffiths et al., 2009; Auer and Griffiths, 2013). The use of pop-up messages that appear on-screen while an individual is gambling on a slot machine and/or online is one way of informing players about how much time they have been playing and/or how much money they have spent. Pop-up messages are one of a range of tools that have been increasingly used by gaming operators to help encourage responsible gambling (Griffiths, 2012). Providing specific information in the form of messages to players while gambling is one way of intervening and helping gamblers that play excessively. It is believed that information that is given to people to enable behavioral change should encourage reflection as research has shown that self-monitoring changes behavior in the desired direction (e.g., Gilberts et al., 2001; Hardeman et al., 2002; Schwedes et al., 2002). However, it remains to be determined whether these pop-up interventions deliver the desired effects among the players that receive such messaging.

### The Use of Pop-up Messages in Gambling

Experimental studies on gamblers playing slot machines (e.g., Monaghan et al., 2009; Monaghan and Blaszczynski, 2010) have shown that giving players messages that encourage self-appraisal (e.g., “*Do you know how long you have been playing? Do you need to think about a break?*”) result in a significantly greater effect on self-reported thoughts during playing sessions and subsequent playing behavior compared to pure informative messages. One study reported that exposure to a warning banner informing players of the randomness of outcomes of video lottery terminal (VLT) games decreased faulty gambling beliefs in both problem and non-problem VLT gamblers (Gallagher et al., 2011).

Pop-up messaging has also been used to help gamblers set limits while gambling. Stewart and Wohl (2013) also showed that adherence to monetary limits was significantly more likely among participants that received a monetary limit pop-up message compared to participants who did not receive the pop-up message. In another study, Wohl et al. (2013) simultaneously investigated two responsible gambling tools that targeted adherence to monetary limits among 72 EGM (electronic gaming machine) players. These tools comprised an animation-based educational video (used previously by Wohl et al., 2010) and a pop-up message. In this experiment, EGM gamblers were required to set a monetary limit before commencing play and half the participants were informed when they had reached their money limit via a pop-up message. Both, single and additive effects in addition to possible linear or non-linear interactions were subject to analysis. Confirming previous findings, both responsible gaming tools showed the anticipated single effects. A monetary pop-up reminder helped gamblers to stay within the preset limits. However, no synergy between the monetary pop-up reminder and the animation-based educational information was found. EGM gamblers that received animation-based information in addition to a monetary pop-up reminder did not adhere to the preset limit more often compared to EGM gamblers that only received a monetary pop-up reminder. Another more recent study from the same team also found that pop-up messages can help gamblers keep within their spending limits (Kim et al., 2014).

Studies have also investigated the optimum time at which pop-up messaging should occur within a gambling session. Ladouceur and Sevigny (2009) reported the most effective social responsibility feature was a pop-up message after 60 min of gambling (compared to 15, 30, and 45 min) and resulted in an overall decrease in the length of time spent gambling among players. Schrans et al. (2004) investigated the benefits of a 30-min pop-up compared to a 60-min pop-up on VLTs. They found that earlier exposure to pop-up messages during gambling did not influence either the likelihood of reading the message or choosing to stop play instead of selecting “yes” to continue. A study by Schellink and Schrans (2002) carried out for the *Atlantic Lottery Corporation* in Canada found out that the 60-min pop-up message was associated with a small reduction in session length and a decrease in expenditure among high risk players. Taken as a whole, these few studies suggest that the optimum time for providing a pop-up message for those who play excessively is after 1 h of continuous gambling.

The preceding literature shows that almost all studies investigating pop-up messages have mainly been conducted in laboratory settings. In a review of the existing literature on pop-up messages Monaghan (2008) emphasized the need for field studies. A study by Auer et al. (2014) investigated the effects of a slot machine pop-up message in a real gambling environment by comparing the behavioral tracking data of two representative random samples of 400,000 gambling sessions before and after the pop-up message was introduced by an online gaming operator. The study comprised approximately 200,000 gamblers of which only a few thousand played sessions comprising 1,000 consecutive games or more. The results indicated that, following the viewing of a pop-up message after 1,000 consecutive gambles on an online slot machine game (i.e., “*You have now played 1,000 slot games. Do you want to continue? [YES/NO]*”), nine times more gamblers (45 out of a few thousand players) ceased their gambling at exactly 1,000 games than did those gamblers who had not viewed the message after playing exactly 1,000 games (5 out of a few thousand players). The authors concluded that pop-up messages influence a very small number of gamblers to cease their playing session.

### Self-Efficacy, Information Giving, and Behavior Change

An important component of any performed behavior is self-efficacy. Self-efficacy reflects the extent to which a person feels capable of performing a behavior and is the focus of social cognitive theory in which individuals learn by observing the behavior of other individuals (Bandura, 2001). Furthermore, self-efficacy is central to almost all information-processing models found in the health communication literature including the Theory of Planned Behavior (Ajzen, 1985), the Health Belief Model (Maiman and Becker, 1974; Janz and Becker, 1984), the Extended Parallel Process Model (Witte, 1992), and Protection Motivation Theory (Rogers, 1983). All of these theories posit that if a message can strengthen self-efficacy beliefs, behavioral change is more likely to happen. More specifically, these theories posit that for information to change behavior, the messages must possess efficacy components, including both self-efficacy (the belief that an individual can do an action) and response efficacy (the belief that a recommended action will have a desired outcome for the individual; Witte et al., 2001; Perloff, 2008). To change a health behavior after exposure to a specific message, individuals must believe there is an action that they are capable of carrying out and that the action will help them adhere to the message (Witte et al., 2001). In any form of persuasive communication with the aim of changing behavior, all of these theories note that it is important to specify which constructs and processes (i) are the most relevant to the target group, (ii) are predictive of the behavior in question, and (iii) can be influenced to promote the desired change in behavior (Donovan and Henley, 2010).

Another potential way of trying to enable behavioral change in gambling is the use of normative feedback. Normative beliefs have significantly influenced the behavioral outcome in studies getting individuals to quit smoking (Van den Putte et al., 2009; Becker et al., 2014), use condoms (Yzer et al., 2000) and reduce marijuana consumption (Yzer et al., 2007). In a study of American college student gambling, Celio and Lisman (2014) demonstrated that personalized normative feedback decreased other students’ perceptions of gambling and lowered risk-taking performance on two analog measures of gambling. They concluded that a standalone personalized normative feedback intervention may modify gambling behavior among college students. In the use of motivational interviewing, Miller and Rollnick (1991) have also emphasized normative feedback as an important aspect in facilitating behavioral change.

### The Present Study

As normative feedback and information to aid self-efficacy appear to be essential aspects in influencing behavioral change, the present authors hypothesized that giving such information to gamblers might influence playing cessation if applied to pop-up messages while gambling. To the authors’ knowledge, self-appraisal feedback (i.e., information that helps an individual reflect on their own gambling behavior), normative feedback (i.e., information that compares an individual’s own gambling behavior with others), cognitive belief feedback (i.e., factual information given to the individual about false gambling beliefs), and self-efficacy feedback (i.e., information that provides help on how they can change their behavior) have never been empirically examined in any real-world online gambling setting. Therefore, the present study investigated the effects of a normative and self-appraisal pop-up message among online slot machine players on a real online gambling site. Using the same methodology as a previously published study (i.e., Auer et al., 2014), the goal of the present study was to investigate whether enhanced content on a pop-up message has any additional effect on player behavior (i.e., will more players stop gambling after seeing an enhanced pop-up message compared to a simple message). It was hypothesized that the enhanced message with enhanced feedback content would lead to an increase in gamblers terminating their gambling session after playing 1,000 consecutive slot games compared to those gamblers who viewed a simple information-based message.

## Materials and Methods

### Background Information and Data Access

The present authors were given access to a large anonymized dataset from a commercial online gambling operator. In 2011, the online gambling operator decided to supplement their responsible gambling features and introduced a simple pop-up message that is triggered if their customers play 1,000 consecutive games (i.e., approximately 1 hour’s play) on slot machines during a single online gambling session. A gambling session is initiated when a player logs into their individual account and is terminated if the player logs out or closes their web-browser. The 1,000-game threshold was the gaming operator’s decision and the authors did not have any influence on when the pop-up message was initiated. The operator’s reason for choosing a threshold of 1,000 slot games was partly based on the findings of previous studies outlined in the introduction that playing 1,000 games takes approximately 1 h (i.e., Schellink and Schrans, 2002; Schrans et al., 2004; Ladouceur and Sevigny, 2009). From a technical perspective, it was also easier for the operator to track the number of games played by the gamblers rather than their overall playing time.

### Details About the Pop-up Message

After the pop-up message has appeared on-screen, the player can then decide whether to stop or to continue the gambling session. The original (“simple”) pop-up message appeared in the center of the screen and simply informed the player that 1,000 games had been played and gave the player the option to continue or to stop gambling. The pop-up remained on the screen until the player pressed “yes” or “no” as to whether they wanted to continue gambling. If the player pressed “yes,” the pop-up message immediately disappeared. If the player pressed “no,” the game window immediately closed. The size of the pop-up was approximately one-eighth of the full screen.

In September 2013, the content of the pop-up message was further enhanced to include self-appraisal, normative feedback and text to address cognitive beliefs commonly found among gamblers, and a recommendation to enhance self-efficacy. The new pop-up message’s content was developed by the present authors, and was enhanced because a previous study (i.e., Auer et al., 2014) noted how limited and simplistic the original message was. The present study compared the adherence to the enhanced pop-up with the adherence to the original pop-up. In order to analyze the effect of the more recently introduced pop-up message, the authors accessed two representative random samples of 800,000 sessions 3 months before and 3 months after the new enhanced pop-up message was introduced. The total dataset comprised 1,600,000 game sessions that contained at least one slot game with approximately 70,000 online slot machine gamblers. The methodology is therefore quasi-experimental as it compares gambling behavior across two different time periods. Data collected in the present study took place between June 2013 and November 2013.

### Details of (and Rationale for) the Enhanced Pop-up Message

The new pop-up message (translated from German, the native language used on the German-speaking site and the native language of one of the present authors) read: “*We would like to inform you, that you have just played 1,000 slot games. Only a few people play more than 1,000 slot games. The chance of winning does not increase with the duration of the session. Taking a break often helps, and you can choose the duration of the break*”^1^. The reasoning behind the messaging is as follows:

– “*We would like to inform you, that you have just played 1,000 slot games*”: This part of the message objectively informs players about the behavior they engaged in.
– “*Only a few people play more than 1,000 slot games*”: This part of the message provides normative feedback that very few other gamblers play 1,000 consecutive slots games.
– “*The chance of winning does not increase with the duration of the session*”: This part of the message addresses a common misbelief among gamblers [i.e., the gamblers’ fallacy (Griffiths, 1994)].
– “*Taking a break often helps, and you can choose the duration of the break*”: This part of the message provides advice (to aid self-efficacy) and leaves the decision up to the player and is in line with the techniques of motivational interviewing (Miller and Rollnick, 1991).

Apart from the content of the message, nothing else in the pop-up was changed across the two conditions (e.g., size, location on the screen, etc.). A player has to press the “*Spiel beenden*” (“*Close game*”) button to exit the playing session. If the player presses the “*OK*” button, the pop-up disappears and the playing session continues. The “*close game*” link and the “*OK*” button were exactly the same in both conditions. This is important with respect to the interpretation of the results. All changes in effectiveness of the message in changing gamblers’ behavior can solely be traced back to changes in message content, as all other variables in the two playing conditions were identical. The study was given ethical approval by the research team’s University Ethics Committee.

### Details of the Dataset and Analytic Strategy

The 800,000 sessions with the original pop-up message comprised 11,232 sessions where at least 1,000 consecutive slot games had been played (1.4% of the total sessions prior to the enhanced message being introduced). The 800,000 sessions with the new enhanced message comprised 11,878 sessions where at least 1,000 consecutive slot games had been played (1.48% of the total sessions after the enhanced message had been introduced). These figures demonstrate that the ratio of the most “highly involved” players was similar in both study conditions and increases the validity of the study. Given the low percentages of sessions that reached 1,000 consecutive plays on the online slot machine, high gaming intensity (i.e., high gambling involvement as defined by the number of consecutive games played) is relatively rare among the player base examined. The authors assumed that the threshold of playing more than 1,000 consecutive slot games per session reliably identified only the most highly involved gamblers. The effectiveness of the pop-up message in both conditions was determined by the number of sessions that terminated after playing 1,000 consecutive slot games. The design was between-subjects, however some (or perhaps even most) of the participants in the original pop-up message condition may have also been in the enhanced message condition as they were all clientele of the same gambling operator. The researchers were given access to two anonymized datasets, therefore it was not possible to calculate how many of the same players participated in both conditions.

## Results

Of the 11,232 sessions that lasted at least 1,000 consecutive slot games and received the original pop-up message, 75 sessions immediately terminated after the pop-up message was shown at the 1,000th consecutive game (0.67%). This behavior cessation was almost certainly due to the appearance of the pop-up message. Of the 11,787 sessions that lasted at least 1,000 consecutive slot games and received the enhanced pop-up message, 169 sessions immediately terminated after the pop-up was shown at the 1,000th game (1.39%).

This percentage of players stopping at 1,000 consecutive slot games was significantly higher than the percentage stopping as a consequence of the original pop-up message [*χ*^2^(1) = 31.51, *p* < 0.001]. However, large sample sizes often lead to significant results and are not necessarily meaningful. For this reason, the effect size was also calculated. With binary outcomes, the effect size can be derived from the Odds Ratio (OR; Chinn, 2000). The OR is computed from the chance of “success” in one group relative to the change of “success” in another group. Table 1 shows the number of players who ceased or continued to play in the pre- and post-condition. The OR is computed as follows: OR = $\frac{\frac{a}{a+b}}{\frac{c}{c+d}}$ if the cells of the contingency table are labeled in a clockwise manner. In this case, the OR was 2.13 = $\frac{\frac{169}{11,878}}{\frac{75}{11,232}}$. Chinn (2000) reports that the natural logarithm of the OR can be converted to Cohen’s *d* (Cohen, 1992), a measure of effect size, by dividing it by 1.81. A Cohen’s *d* value of 0.42 results when applying the formula $\frac{\text{In}\left(\text{OR}\right)}{1.81}$. Values between 0.2 and 0.5 are regarded as being small effect sizes (Cohen, 1992). The results therefore show there is an effect. However, the effect is modest.

**TABLE 1:** **Contingency table showing the number of players who stopped after playing 1,000 consecutive games on an online slot machine during the pre-condition (original pop-up message) and post-condition (enhanced pop-up message)**.

	**Ceased to play**	**Continued to play**	**Total**
Post-condition	169	11,709	11,878
Pre-condition	75	11,157	11,232
Total	244	22,886	23,110

**FIGURE 1:**
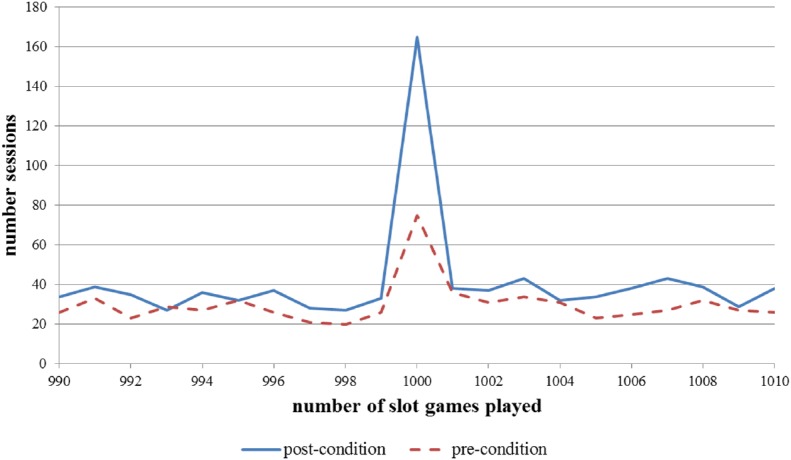
**Number of sessions that lasted exactly 990 to 1,010 consecutive games on an online slot machine during the pre-condition (original pop-up message) and post-condition (enhanced pop-up message)**.

The effect is further highlighted by Figure 1 that shows a clear visible spike that only appears when the pop-up message is shown (i.e., at the playing of 1,000 consecutive slot games). The x-axis range between sessions lasting 990 games to sessions lasting 1,010 games was chosen purely for visual presentation purposes. The selection of this range highlights the spike at exactly 1,000 games played, whereas the number of sessions ending at slightly less than 1,000 games or slightly more than 1,000 games is fairly similar. Figure 1 shows the effect of the pop-up is clearly visible, both before and after the message was changed. However, the effect is greater after the original (simple) pop-up message was changed to the enhanced one.

In the original pop-up message condition, the 75 sessions that immediately ceased after 1,000 consecutive slot games were produced by 71 different players (95%). This demonstrates that very few players reacted to the pop-up message more than once. In the enhanced pop-up message condition, the 169 sessions that immediately ceased after 1,000 consecutive slot games were produced by 139 different players (84%). This also demonstrates that few players reacted to the pop-up more than once. However, the percentage of players who stopped gambling after viewing the pop-up message more than once was higher in the enhanced pop-up message condition. This suggests that the enhanced pop-up message encouraged more players to make use of it more often compared to the original pop-up message.

On the other hand, results showed that 59 of the 71 different players (83%) that terminated their sessions before the pop-up was changed ignored the pop-up message at least once in another session and played more than 1,000 slot games within one session. After the pop-up was enhanced, the number of players that ignored the pop-up message at least once was 98 out of 139 (71%). The percentage of players that ignored the pop-up message was lower if informed by an enhanced pop-up compared to the purely informative pop-up message. This means that players that made use of the enhanced pop-up message were less likely to ignore it at other times compared to the purely informative pop-up message. This difference was significant [*χ*^2^(1) = 3.95, *p* < 0.05]. However, the OR was 1.18 (Cohen’s *d* effect size = 0.09), and is therefore negligible according to Cohen (1996).

## Discussion

The current study utilized an empirical sample of 1.6 million game sessions (comprising approximately 70,000 online slot machine gamblers) and provided ecologically reliable behavioral information on the effectiveness of pop-up messaging while gambling. Consequently, the data are truly objective and not subject to the recall bias effects of self-report methods or the lack of ecological validity in laboratory experiments. The effectiveness of two different types of pop-up message was examined and showed that enhanced pop-up messages led to 1.39% of highly involved gamblers immediately ceasing their gambling session compared to 0.67% of highly involved gamblers that only saw the simple pop-up messaging. As the two spikes in Figure 1 demonstrate, the cessation of the playing sessions was almost certainly due to viewing the pop-up message. The percentage of players that immediately terminated their sessions due to the viewing of the pop-up message doubled from 0.67 to 1.39% as a consequence of enhancing the message with self-appraisal, normative, and cognitive belief content (compared to self-appraisal only). All other aspects of the pop-up message were identical in the two conditions. This difference was not only statistically significant but also meaningful as demonstrated by the modest effect size.

It should be acknowledged that the current study is not a true experiment as the participants were not the same in the two conditions (however, it is likely there would be a large overlap as the data were collected from the same gaming company’s customer base within a short time period). The study would best be described as quasi-experimental in that a pre-condition was compared to a post-condition across different points in time. The present authors are not aware of any significant changes in the gambling operator’s environment during the 6 months of the research period. The percentage of sessions that lasted at least 1,000 consecutive slot games was roughly the same during the pre-condition and the post-condition period. In the original pop-up condition, only 1.4% of the 800,000 sessions (*n* = 11,232) lasted longer than 1,000 consecutive slot games. In the enhanced pop-up condition, only 1.48% of the 800,000 sessions (*n* = 11,878) lasted longer than 1,000 consecutive slot games. The similarity in percentages supports the claim of overall unchanged conditions, both before and after the pop-up was enhanced. If there was a significant difference in these percentages, one could question the validity of the study because important conditions (e.g., the nature of the games, promotional activity, playing behavior, etc.) could have changed.

All assumptions made by the present authors in a previously published study (i.e., Auer et al., 2014) also hold true for the present study because the follow-up study was conducted in the same real-world setting. Auer et al. (2014) concluded that the results they obtained appeared to show that the introduction of a mandatory pop-up message had a small effect in stopping gambling behavior among a small number of gamblers. In that study, nine times more gamblers ceased their gambling session following the viewing of a pop-up message after 1,000 consecutive gambles on an online slot machine game compared to those who had not viewed a pop-up message at all. In the present study, twice as many gamblers ceased to gamble when presented with an enhanced pop-up message compared to the simple pop-up message. This enhanced pop-up contained normative, self-appraisal, and cognitive-belief content as well as behavioral advice to aid self-efficacy. All these aspects have been argued to influence gambling behavior and enable behavioral change (Auer and Griffiths, 2014), but have never been tested in an empirical setting. To the authors’ knowledge, the changing and comparison of textual content in pop-up messaging has never previously been subject to empirical research.

To date, very few studies have been published that empirically investigate effectiveness of social responsibility tools in real world settings. This study adds to the sparse empirical base both generally and in relation to pop-up messaging more specifically. Previous research has often relied on self-report or experimental data, often in laboratory settings, to investigate the effects of pop-up messages on behavioral and/or cognitive processes such as belief patterns or dissociative states. Although such work is valid and important, laboratory study samples are typically much smaller than other methodologies (e.g., surveys, behavioral tracking studies) and behavioral results in laboratory situations can be distorted by the non-ecological validity of these artificial settings.

There are, of course, limitations to the data collected. Gainsbury and Blaszczynski (2011) suggested using both methodologies (i.e., laboratory and field) to test hypotheses. Therefore, caution should be taken in interpreting results when only one approach or methodology was used. The present authors did not have access to any other information about the samples (e.g., age, sex, income, ethnicity, levels of pathology) so it is not known if the groups in the two conditions differed on any key variables. Another important limitation to the present study was that it was cross-sectional and quasi-experimental in design. As such, the gamblers were not necessarily the same during the pre- and post- pop-up message intervention and this may be a significant limitation for interpretation of the results. However (as mentioned previously), there is no evidence to suggest that the most heavily involved gamblers before and after the change in pop-up messaging did not comprise many of the same people as these were all presumably regular gamblers on this particular website and the study’s data were collected over a relatively short time period (i.e., 6 months).

Although the message in the present study was enhanced with text based on psychological theory relating to behavior change, it cannot be determined which specific aspect(s) (i.e., normative, self-appraisal, cognitive-belief and/or information to aid self-efficacy) had the greatest effect in enabling the small behavioral change. The additional benefit may also be due to the fact that the enhanced message was simply much longer than the previous message text. It is also worth noting that the normative part of the pop-up message was a general statement (“*Only a few people play more than a 1,000 games*”). A much more specific statement may have had a more pronounced effect on the results. If the present study was replicated, it could perhaps include a second pop-up asking players to specifically indicate why they had stopped on seeing the enhanced pop-up (i.e., asking them which part or parts of the message were the most effective in determining cessation of play). Alternatively, an experimental study in which every different permutation is applied with more specific messages could be designed. Such an approach would also shed light on possible synergies and interactions between the different intervention strategies, much like the research of Wohl et al. (2013). However, the underlying study was conducted in a real-world gambling environment and ecological validity was therefore much higher than a laboratory study.

Overall, the data suggest that pop-up messages influence only a small number of gamblers to cease long playing sessions and that enhanced messages are slightly more effective in helping gamblers to stop playing in-session. It is the present authors’ contention that the most likely explanation for the doubling of sessions stopping in the enhanced feedback condition was due to the changed content of the pop-up message. Looking at the results, some may argue that the findings show that pop-up messages are ineffective in changing the behavior of a high-intensity gambler (as only 0.67 to 1.39% across the two conditions ceased gambling). However, the present authors take a more optimistic view in that pop-up messages are only one of a range of responsible gambling tools that are available, and that that the additive effect of such a feature when combined with other responsible gambling features available (e.g., time and money spending limits, self-exclusion options, etc.) is of use. Also, the often-said maxim of “even one problem gambler is one too many” suggests that pop-ups do help some gamblers—even if it is a very small minority.

Taking the more optimistic line about the results presented here, future studies should try to determine the specific impacts of different theoretical concepts such as normative beliefs, self-appraisal, and information that aids self-efficacy. Ultimately it will be gaming operators that implement responsible gaming initiatives. Real world studies such as the present one are an important way of determining the practical effectiveness of pop-up interventions. At present, several responsible gambling accreditation organizations (e.g., *GamCare*) mandatorily require pop-ups, and this is another reason to investigate their impact in real world environments. However, it has to be emphasized that real world studies are accompanied with specific strengths as well as specific weaknesses. The main strength is the high external validity, because the intervention occurred in a real world setting and the study participants were real players. On the other hand, external factors cannot be controlled in the same manner as in laboratory-based studies. Overall, the findings presented here provide a potentially important insight into the effectiveness (or non-effectiveness depending upon viewpoint) of pop-up messaging as a responsible gambling intervention for gaming operators around the world that provide screen-based games.

### Conflict of Interest Statement

The authors declare that the research was conducted in the absence of any commercial or financial relationships that could be construed as a potential conflict of interest.

## Footnotes

1 One of the previous reviewers noted that the enhanced message as it was worded may have given the gambler the impression that taking a break from gambling would help increase the chances of winning. While this is theoretically possible, the authors think this is highly unlikely that the gamblers would have thought this given the content of the message as a whole, and because the message was in German rather than the English translation provided in this paper.
